# Arsenic exposure during pregnancy and postpartum maternal glucose tolerance: evidence from Bangladesh

**DOI:** 10.1186/s12940-021-00811-1

**Published:** 2022-01-14

**Authors:** Abby F. Fleisch, Sudipta Kumer Mukherjee, Subrata K. Biswas, John F. Obrycki, Sheikh Muhammad Ekramullah, D. M. Arman, Joynul Islam, David C. Christiani, Maitreyi Mazumdar

**Affiliations:** 1 Pediatric Endocrinology and Diabetes, Maine Medical Center, Portland, ME USA; 2 Center for Outcomes Research and Evaluation, Maine Medical Center Research Institute, Portland, ME USA; 3 Department of Paediatric Neurosurgery, National Institute of Neurosciences & Hospital, Dhaka, Bangladesh; 4 Department of Biochemistry and Molecular Biology, Bangabandhu Sheikh Mujib Medical University, Dhaka, Bangladesh; 5 Department of Neurology, Boston Children’s Hospital, 300 Longwood Ave, Boston, MA 02115 USA; 6 Department of Environmental Health, Harvard T.H. Chan School of Public Health, Boston, USA

**Keywords:** Pregnancy, Arsenic, Postpartum, Insulin resistance, Beta cell function, Bangladesh

## Abstract

**Background:**

Arsenic exposure has been associated with gestational diabetes mellitus. However, the extent to which arsenic exposure during pregnancy is associated with postpartum glucose intolerance is unknown.

**Methods:**

We studied 323 women in Bangladesh. We assessed arsenic exposure in early pregnancy via toenail and water samples. We measured fasting glucose and insulin in serum at a mean (SD) of 4.0 (3.5) weeks post-delivery. We ran covariate-adjusted, linear regression models to examine associations of arsenic concentrations with HOMA-IR, a marker of insulin resistance, and HOMA-β, a marker of beta cell function.

**Results:**

Median (IQR) arsenic concentration was 0.45 (0.67) μg/g in toenails and 2.0 (6.5) μg/L in drinking water. Arsenic concentrations during pregnancy were not associated with insulin resistance or beta cell function postpartum. HOMA-IR was 0.07% (− 3.13, 3.37) higher and HOMA-β was 0.96% (− 3.83, 1.99) lower per IQR increment in toenail arsenic, but effect estimates were small and confidence intervals crossed the null.

**Conclusions:**

Although arsenic exposure during pregnancy has been consistently associated with gestational diabetes mellitus, we found no clear evidence for an adverse effect on postpartum insulin resistance or beta cell function.

**Supplementary Information:**

The online version contains supplementary material available at 10.1186/s12940-021-00811-1.

## Background

Arsenic, which is naturally-occurring, contaminates the groundwater of many countries, including Bangladesh, Chile, China, India, Mexico, and the United States [1]. Chronic exposure to arsenic in drinking water has been associated with greater risk of adverse health outcomes such as cardiovascular disease and skin, bladder, and lung cancer [2].

Arsenic exposure has also been consistently associated with glucose intolerance and type 2 diabetes in numerous adult cohorts (reviewed in [3, 4]). In vitro and rodent models suggest that arsenic impairs glucose homeostasis by acting to both increase insulin resistance and decrease insulin secretion [3, 5, 6]. Specifically, arsenic accumulates in the pancreas and increases oxidative stress through production of reactive oxygen species which can directly damage pancreatic islet cells [7–9]. Arsenic can also interfere with gene expression (i.e., enhances expression of TNF-α, and IL-6 and inhibits expression of PPAR-γ) to directly increase insulin resistance [10–12]. Consistent with these mechanistic underpinnings, studies of arsenic exposure with biochemical assessment of glucose homeostasis in non-pregnant adults have shown associations with greater insulin resistance [13, 14] and lower insulin secretion [14–17].

There is also a burgeoning population-based literature linking arsenic exposure during pregnancy with gestational diabetes mellitus (GDM) [17–26]. However, to our knowledge, these studies have all assessed the clinical outpoint of GDM rather than the mechanistically relevant biochemical markers of insulin resistance and insulin secretion. Also, the prior studies did not examine glycemia in the postpartum period. Characterizing the role of arsenic exposure on glucose tolerance post-delivery is important because glycemic status in the months postpartum is a more sensitive indicator of future type 2 diabetes risk than the diagnosis of GDM itself [26].

Here, we present an analysis of prenatal arsenic exposure (measured in toenail sample and in home water source) and *postpartum* insulin resistance and beta cell function [measured mean (SD) 4.0 (3.5) months post-delivery] in an arsenic-exposed population in Bangladesh. We hypothesized that women with greater arsenic exposure would have greater insulin resistance and lower insulin secretion.

## Methods

### Study population and design

In this analysis, we leveraged data collected starting in December 2016 as part of a case-control study of arsenic exposure and neural tube defects in Bangladesh [27]. There was high-level arsenic contamination across Bangladesh due to naturally-occurring arsenic sediment which contaminated the water in the tubewells that Bangladeshi citizens relied on for drinking water. Over the past two decades, the Bangladeshi government and nongovernmental organizations have successfully lowered citizens’ arsenic body burden by installing water filtration devices or color-coding unsafe tubewells [28]. Despite this remediation, arsenic exposure levels in Bangladesh at the time of this study were still orders of magnitude higher than in arsenic-exposed areas of the US [20]. One unique feature of Bangladesh is the wide range of arsenic exposures within the country [29].

We reviewed medical records from the National Institute of Neurosciences and Hospital (NINS) in Dhaka to identify mothers who had presented to the hospital for evaluation of a child with myelomeningocele or meningocele (cases). NINS is a government hospital that draws patients from across the country. We recruited controls from NINS or Dhaka Shishu Hospital, adjacent to NINS. We required controls to have delivered an infant within 6 months of a case and required controls to use a different tube well water source than any of the cases. At study enrollment [mean (SD) 4 (3.5 months) postpartum], trained medical staff confirmed presence (cases) or absence (controls) of a neural tube defect.

The present analysis of arsenic exposure and glucose homeostasis uses data from the full cohort, adjusted for case status. At the time of this analysis, we had data available on 365 women. We excluded 11 participants with pre-existing diabetes, 30 without measures of insulin or glucose, and 1 without a measure of arsenic exposure. In total, we studied 323 participants (164 cases and 159 controls). Our analytic sample size ranged from 320 to 323 depending on the exposure and outcome; one participant was missing a measure of toenail arsenic and was excluded from analyses that involved toenail arsenic. Two participants had a negative HOMA-β related to low glucose and were excluded from analyses that involved HOMA-β. We obtained written informed consent from all participants, and the study was approved by the Human Research Committees at Boston Children’s Hospital (BCH), NINS, and the Bangladesh Medical Research Council.

### Measurement of arsenic exposure

We assessed environmental arsenic exposure in two ways, via toenail and water samples [30]. We collected toenail samples from the mother at enrollment, using stainless steel scissors, and we stored samples at room temperature. Samples were analyzed at the Dartmouth Trace Element Core facility using standard total acid digestion procedures [31]. All toenail arsenic values were above the limit of detection (LOD) (0.002 μg/g).

We also collected arsenic samples from the tube well that each participant retrospectively identified as her primary source of drinking water at the time when she became aware of her pregnancy. Because 42% of participants (*n* = 135) changed their drinking water source at some point during their pregnancy, we examined drinking water arsenic concentrations in a secondary analysis. The Environmental Engineering Laboratory at the Bangladesh University of Engineering and Technology (BUET) analyzed water arsenic concentrations using graphite furnace atomic absorption spectroscopy (SM 3113B) [32]. Twenty-eight percent of samples had a water arsenic concentration below the LOD of 1 μg/L, consistent with other studies from the region which has undergone arsenic remediation following groundwater contamination [29]. For samples below the LOD, we estimated water arsenic concentrations to be LOD/2.

### Measurement of insulin resistance and beta cell function

We collected blood samples from participants at enrollment. The Clinical Biochemistry laboratory of Bangabandhu Sheikh Mujib Medical University quantified insulin concentrations using a chemiluminescent microparticle immunoassay on a Ci4100 ARCHITECT plus instrument (Abbott) and NINS clinical laboratory measured glucose concentration using an enzymatic and photometric method. We estimated insulin resistance by calculating the homeostatic model assessment of insulin resistance (HOMA-IR) as [(fasting glucose (mmol/L) × fasting insulin (μU/mL))/22.5]. We estimated beta cell function by calculating the homeostatic model assessment of beta cell function (HOMA-β) as [(20*fasting insulin (μU/mL))/(fasting glucose (mmol/L) – 3.5)].

### Measurement of covariates

We collected information on participant age, education, employment, spouse employment, prenatal betel nut use, prenatal smoking, medications during pregnancy (including insulin use), and birth order of the index child during interviews at study enrollment. We assessed intake of rice and fish as part of a food frequency questionnaire previously validated in rural Bangladeshi populations [33]. We recorded information about the blood draw, including timing (months postpartum) and hours fasting.

### Statistical analyses

We first fit unadjusted, followed by covariate-adjusted, linear regression models to examine the associations of toenail (primary analysis) and drinking water (secondary analysis) arsenic concentrations with HOMA-IR and HOMA-β. We fit separate linear regression models for each outcome. We ln-transformed HOMA-IR and HOMA-β to meet model assumptions. For ease of interpretation, we exponentiated regression coefficients and reported results as a percent change [% change = (exp (beta) – 1) × 100]. We expressed continuous associations per IQR increment in exposure. We adjusted for covariates potentially associated with arsenic exposure [30, 34–37] and/or glucose tolerance [38–40]. We adjusted for age at enrollment (continuous), prenatal betel nut use (yes or no), education (college, high school or less, or no formal schooling), employment (employed or unemployed), spouse employment [unemployed, office worker (small business owner or private office worker), agricultural laborer or carpenter, or unknown], study group (infant with neural tube defect or control), rice intake [cups per day (continuous)], fish intake [cups per day (continuous)], and characteristics of the blood draw [months postpartum (continuous) and fasting time (continuous)] (Supplemental Fig. 1). Adjustment for prenatal smoking status (2 participants smoked during pregnancy) and birth order of the index child (46% of the index children were the family’s first child) did not appreciably change results, and thus we did not include these covariates in final models.

Next, we performed sensitivity analyses using our final adjusted model. We ran the analyses after excluding one participant with a fasting time of less than 5 h, one participant with an implausible glucose concentration (< 50 mg/dL), and 2 participants who used insulin during pregnancy. We also fit models to test the associations of water arsenic concentrations with HOMA-IR and HOMA-β in the subset of participants (*n* = 188) who reported a constant water source during pregnancy. Finally, we fit covariate-adjusted penalized spline generalized additive models to visually examine potential non-linear associations of arsenic concentrations with HOMA-IR and HOMA-β.

We used R 4.0.1 (Vienna, Austria) for all analyses.

## Results

Of the 323 participants included in the analytic dataset, mean (standard deviation [SD]) age at the time of enrollment was 24.3 (4.7) years. Fifty-four percent of participants had high school or less education and 13% used betel nut prenatally. Median (IQR) HOMA-IR was 1.2 (1.2) and HOMA-β was 84.5 (70.1) (Table 1). Median (IQR) postpartum toenail arsenic concentration was 0.45 (0.67) μg/g and drinking water arsenic concentration was 2.0 (6.5) μg/L. Toenail and drinking water arsenic concentrations were moderately correlated (Spearman’s r = 0.44). As compared to participants with lower toenail arsenic concentrations, participants with higher concentrations had greater betel nut use, greater rice intake, and were more likely to have a spouse who was an office worker (rather than an agricultural laborer or carpenter) (Supplemental Table 1).

**Table 1:** Characteristics of participants overall (*n* = 323) and by toenail arsenic concentration (μg/g) (*n* = 322)

		--------------Quartiles of Toenail Arsenic^a^---------------			
	Overall (*n* = 323)	Q1 (*n* = 82)	Q2 (*n* = 79)	Q3 (*n* = 80)	Q4 (*n* = 81)
	Median (IQR) or %	Median (IQR) or %			
**Characteristics**					
Child with neural tube defect (%)	51	52	54	44	52
Age (years)	24.0 (7.0)	25.0 (8.0)	23.0 (7.0)	23.0 (6.2)	24.0 (8.0)
Prenatal betel nut use (%)	44	40	41	42	52
Education					
No formal schooling (%)	20	16	18	28	19
High school or less (%)	54	55	53	51	56
College/University (%)	26	29	29	21	26
Unemployed (%)	96	90	97	99	96
Spouse occupation					
Unemployed (%)	1	1	1	1	0
Office worker (%)	43	37	46	42	49
Agricultural laborer or carpenter (%)	43	49	51	38	35
Unknown (%)	13	13	3	19	16
Rice Intake (cups/day)	6.0 (3.0)	6.0 (5.0)	6.6 (3.0)	6.0 (3.0)	8.0 (5.6)
Fish Intake (cups/day)	0.4 (0.3)	0.5 (0.3)	0.4 (0.3)	0.4 (0.3)	0.4 (0.3)
**HOMA measurement**					
HOMA-IR	1.2 (1.1)	1.2 (1.0)	1.2 (0.9)	1.1 (1.4)	1.1 (1.2)
HOMA-ß	84.5 (70.2)	88.9 (68.8)	81.7 (89.1)	89.7 (67.0)	84.8 (67.2)
Hours fasting	10.9 (5.7)	10.8 (5.2)	11.1 (6.3)	11.3 (4.6)	10.8 (5.8)
Months postpartum	3.1 (6.2)	2.1 (4.5)	3.0 (6.2)	4.6 (7.2)	3.7 (5.7)

^a^Quartile ranges (μg/g): Q1: 0.091-0.278, Q2: 0.279-0.452, Q3: 0.453-0.951, Q4: 0.952-12.299

In unadjusted and covariate-adjusted models, we found no association between arsenic concentrations during pregnancy and postpartum insulin resistance or beta cell function. Participants with greater toenail and water arsenic had higher insulin resistance (HOMA-IR) and lower beta cell function (HOMA-β), although effect estimates were very small and confidence intervals crossed the null. In covariate-adjusted models, for each IQR increment in toenail arsenic, HOMA-IR was 0.07% (− 3.13, 3.37) higher and HOMA-β was 0.96% (− 3.83, 1.99) lower. For each IQR increment in water arsenic, HOMA-IR was 0.31% (− 0.82, 1.46) higher and HOMA-β was 0.09% (− 1.11, 0.94) lower (Table 2).

**Table 2:** Percent change (95% CI) in HOMA-IR or HOMA-β per IQR increment in toenail or water arsenic concentration^a^

	***HOMA-IR***
Toenail Arsenic	0.13 (−3.07, 3.43)
Water Arsenic	0.33 (−0.81, 1.47)
	***HOMA-*β**
Toenail Arsenic	−0.89 (−3.76, 2.06)
Water Arsenic	−0.07 (−1.09, 0.96)

^a^Adjusted for neural tube defect (NTD) group, age, betel nut use, education, spouse occupation, rice intake, fish intake, and blood draw characteristics (hours fasting and months postpartum)

The associations of toenail and water arsenic concentrations with HOMA-IR and HOMA-β were not appreciably different when we excluded 4 participants for short fasting time, implausible glucose concentration, and use of insulin during pregnancy (data not shown). In the subset of participants who reported a constant water source during pregnancy, for each IQR increment in water arsenic, HOMA-IR was 0.12% (− 1.41, 1.66) higher and HOMA-β was 0.02% (− 1.33, 1.31) lower, although confidence intervals crossed the null.

**Fig. 1:**
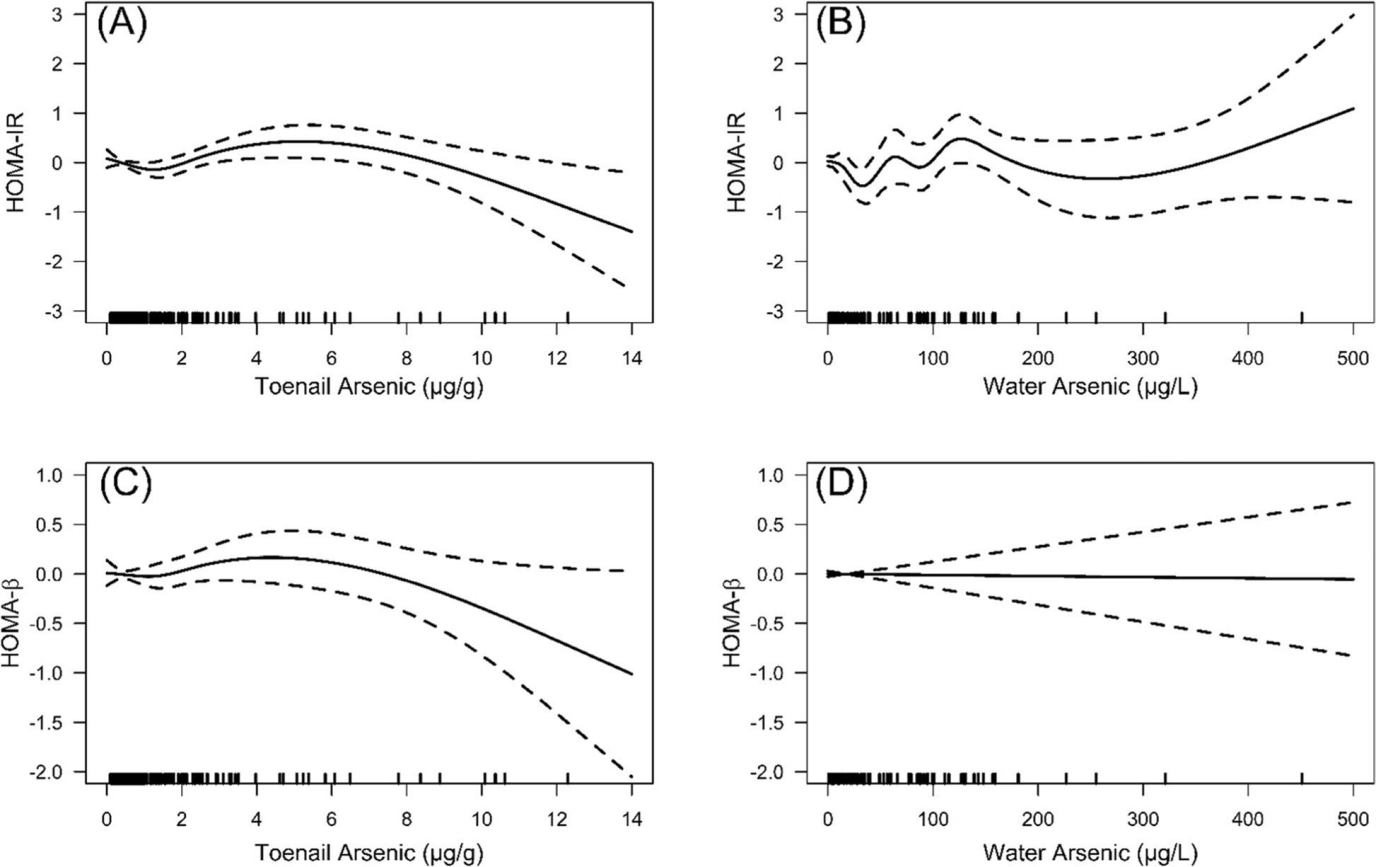
Covariate-adjusted^a^ generalized additive models showing associations of toenail and water arsenic concentrations with insulin resistance (HOMA-IR) and beta cell function (HOMA-β)

In penalized spline generalized additive models, among the majority of participants (arsenic concentrations < 5 μg/g for toenail and < 150 μg/L for water), higher toenail and water arsenic concentrations appeared to be associated with higher HOMA-IR (i.e., worse insulin resistance), and higher toenail arsenic appeared to be associated with higher HOMA- β (improved beta cell function). Among the few participants with very high toenail arsenic concentrations (> 5 μg/g), higher arsenic appeared to be associated with lower HOMA-IR and HOMA- β (i.e., improved insulin resistance but worse beta cell function). Among participants with high water arsenic concentrations (> 150 μg/L) there did not appear to be an association between arsenic and HOMA-IR, and water arsenic concentrations across the range of exposure did not appear to be associated with HOMA- β (Fig. 1).

## Discussion

In a cohort in Bangladesh, we found no association between arsenic exposure during pregnancy and postpartum glucose homeostasis. While women with greater arsenic exposure had greater insulin resistance and lower insulin secretion, effect sizes were small and confidence intervals crossed the null.

To put our results in context of the existing literature, several prior studies [17–26] conducted in regions across a range of arsenic exposures suggest that women with greater arsenic exposure are more likely to develop GDM during pregnancy. A strength of the present study is our toenail measures of arsenic, as the majority of prior studies only measured arsenic in the urine [17, 18, 22] or blood [24–26]. Due to the short (10 h) biological half-life of arsenic, toenail measures which approximate exposure during the 6-12 months prior to collection (i.e., reflecting exposures during early to mid-pregnancy in the present study) provide a more integrated, long-term exposure measure than urine or blood [41, 42]. Another strength of our study is our direct biochemical measures of insulin and glucose. As compared to the prior studies of arsenic exposure during pregnancy which relied on a clinical diagnosis of GDM, we were positioned to detect a more subtle impact of arsenic exposure on insulin resistance and insulin secretion.

Despite our measurement of long-term arsenic exposure and direct biochemical measures of insulin and glucose, in contrast to the prior studies, the association between arsenic exposure and glucose homeostasis did not reach statistical significance in our study. We hypothesize that the impact of arsenic exposure on glucose homeostasis during pregnancy may not persist postpartum, consistent with findings from a recent rodent study [43]. If replicated in other cohorts, this has important clinical implications; while arsenic exposure during pregnancy may increase risk of GDM and associated adverse maternal and fetal perinatal health outcomes, its impact on postpartum glucose tolerance and future risk of type 2 diabetes may be more limited. However, it is possible that the relatively young age of our cohort [mean (SD) 24 (7) years] may have limited our ability to see an association between arsenic exposure and GDM, which is more common in older women [40]. Our wide confidence intervals and the fact that the directionality of our effect estimates was consistent with our a priori hypotheses suggest that we also may have been limited by our relatively small sample size (*N* > 1000 in the majority of the prior studies of arsenic exposure and GDM [17, 18, 20–22, 24, 26]). Thus, our findings warrant replication in larger cohorts of diverse women.

Another novel aspect of our study is that we used spline models to investigate potential non-linear associations between arsenic exposure and postpartum glucose homeostasis. We hypothesized that we might observe protective effects of arsenic on HOMA-IR in women with the highest arsenic concentrations. This was based on recent mouse models that have shown that at higher levels of exposure, arsenic may improve insulin resistance which may offset arsenic-induced impairment in beta-cell function [44–46]. Our spline models were somewhat consistent with these rodent data. Among the women with the highest toenail concentrations of arsenic, higher arsenic exposure was associated with worse beta-cell function but lower (i.e., improved) insulin resistance.

Strengths of our study include two measures of arsenic exposure during pregnancy including toenail measures of arsenic exposure and *postpartum* biochemical measures of both insulin resistance and beta cell function. Limitations of our study include a relatively small sample size and no information on pre-pregnancy body mass index.

## Conclusion

In summary, in a Bangladeshi cohort, we found no clear evidence for an adverse effect of arsenic exposure during pregnancy on postpartum insulin resistance or beta cell function. If replicated in other cohorts, our finding has implications for surveillance for abnormal glucose homeostasis among women in arsenic endemic areas of the world.

## Supplementary Information

**Additional file 1.**

## Data Availability

The datasets used and/or analyzed during the current study are available from the study team (maitreyi.mazumdar@childrens.harvard.edu) upon reasonable request.

## Abbreviations

CI: Confidence interval
IQR: Interquartile range
LOD: Limit of detection
GDM: Gestational diabetes mellitus
SD: Standard deviation
HOMA-IR: Homeostatic model assessment of insulin resistance
HOMA-β: Homeostatic model assessment of beta cell function
