# Supplementary material for: Arsenic exposure during pregnancy and postpartum maternal glucose tolerance: evidence from Bangladesh
Source: Environ Health. 2022 Jan 14;21:13. doi: 10.1186/s12940-021-00811-1 (PMC8759206; doi:10.1186/s12940-021-00811-1)
Supplement: Supplementary file 1 — Additional file 1. [file 12940_2021_811_MOESM1_ESM.docx]

**Supplemental Table 1**. Characteristics of participants by water arsenic concentration (µg/L)

|  | --------------Quartiles of Water Arsenic^a^--------------- | | | | |
| --- | --- | --- | --- | --- | --- |
|  | Q1  (n=92) | Q2  (n=84) | Q3  (n=76) | Q4  (n=71) |  |
|  | Median (IQR) or % | | | | |
| **Characteristics** |  |  |  |  |  |
| Child with neural tube defect (%) | 46 | 56 | 49 | 54 |  |
| Age (years) | 23.0 (7.0) | 24.0 (7.0) | 25.0 (6.2) | 23.0 (8.0) |  |
| Prenatal betel nut use (%) | 37 | 40 | 47 | 54 |  |
| Education |  |  |  |  |  |
| No formal schooling (%) | 20 | 20 | 16 | 24 |  |
| High school or less (%) | 49 | 54 | 64 | 49 |  |
| College/University (%) | 32 | 26 | 20 | 27 |  |
| Unemployed (%) | 92 | 96 | 97 | 97 |  |
| Spouse occupation |  |  |  |  |  |
| Unemployed (%) | 2 | 0 | 1 | 0 |  |
| Office worker (%) | 40 | 49 | 37 | 48 |  |
| Agricultural laborer or carpenter (%) | 48 | 43 | 47 | 32 |  |
| Unknown (%) | 10 | 8 | 14 | 20 |  |
| Rice Intake (cups/day) | 6.2 (3.0) | 6.0 (3.0) | 6.0 (6.0) | 9.0 3.0) |  |
| Fish Intake (cups/day) | 0.5 (0.3) | 0.4 (0.2) | 0.4 (0.3) | 0.4 (0.3) |  |
|  |  |  |  |  |  |
| **HOMA measurement** |  |  |  |  |  |
| HOMA-IR | 1.4 (1.2) | 1.0 (1.1) | 1.2 (0.8) | 1.0 (1.5) |  |
| HOMA-ß | 107.4 (81.6) | 70.2 66.4) | 90.7 (56.1) | 79.3 (77.8) |  |
| Hours fasting | 10.5 (6.4) | 11.0 (6.1) | 11.7 (3.2) | 10.4 (5.4) |  |
| Months postpartum | 3.1 (6.2) | 2.1 (6.3) | 4.0 (6.8) | 3.1 (5.0) |  |

^a^ Quartile ranges (µg/L): Q1: < LOD (1.0), Q2: 1.0 - 2.0, Q3:3.0 - 7.0, Q4: 8.0 – 451.0

**Supplemental Figure 1.** Conceptual model of variables accounted for in our analysis of arsenic exposure during pregnancy with postpartum insulin resistance and beta cell function.

**
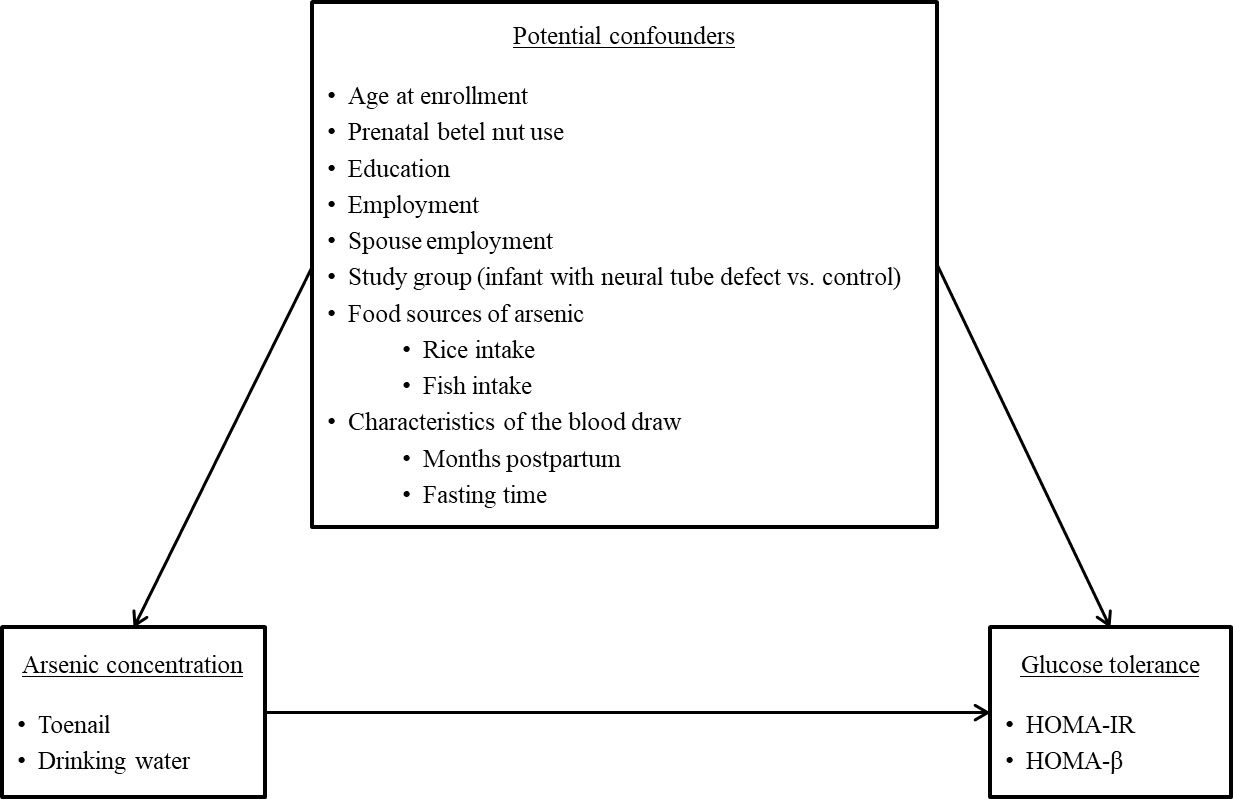
**
